# Impacts of external rotators and the ischiofemoral ligament on preventing excessive internal hip rotation: a cadaveric study

**DOI:** 10.1186/s13018-021-02873-w

**Published:** 2022-01-04

**Authors:** Kazuyoshi Baba, Daisuke Chiba, Yu Mori, Yoshiyuki Kuwahara, Atsushi Kogure, Takehiro Sugaya, Kumi Kamata, Itsuki Oizumi, Takayuki Suzuki, Hiroaki Kurishima, Soshi Hamada, Eiji Itoi, Toshimi Aizawa

**Affiliations:** 1 Department of Orthopedic Surgery, Tohoku University School of Medicine, 1-1 Seiryo-machi, Aoba-ku, Sendai, Miyagi 980-8574 Japan; 2 Sendai City Hospital, 1-1-1 Asuto Nagamachi, Taihaku-ku, Sendai, Miyagi 982-8502 Japan; 3 Iwaki Medical Center, 16 Kusehara, Uchigo Mimayamachi, Fukushima, Iwaki 973-8555 Japan; 4 Tohoku Rosai Hospital, 4-3-21, Dainohara, Aoba-ku, Sendai, Miyagi 981-8563 Japan

**Keywords:** External rotator, Capsular ligament, Dislocation, Ischiofemoral ligament, Total hip arthroplasty, Cadaveric study, Hip joint

## Abstract

**Background:**

This study examined the biomechanics of preventing excessive internal hip joint rotation related to the hip flexion angle.

**Method:**

An intramedullary nail with a circular plate equipped with a protractor was installed in the femur of nine normal hips. The circular plate was pulled by 3.15 Nm of force in the internal rotation direction. The external rotators were individually resected, finally cutting the ischiofemoral ligament. The cutting order of the external rotators differed on each side to individually determine the internal rotation resistance. The external rotators were resected from the piriformis to the obturator externus in the right hips and the reverse order in the left hips. Traction was performed after excising each muscle and ischiofemoral ligament. Measurements were taken at 0°, 30°, and 60° of hip flexion, and the differences from baseline were calculated.

**Results:**

For the right hip measurements, the piriformis and ischiofemoral ligament resection significantly differed at 0° of flexion (p = 0.02), each external rotator and the ischiofemoral ligament resections significantly differed at 30° of flexion (p < 0.01), and the ischiofemoral ligament and piriformis and inferior gemellus resections significantly differed at 60° of flexion (p = 0.04 and p = 0.02, respectively). In the left hips, the ischiofemoral ligament and obturator externus, inferior gemellus, and obturator internus resections significantly differed at 0° of flexion (p < 0.01, p < 0.01, and p = 0.01, respectively), as did each external rotator and the ischiofemoral ligament resections at 30° of flexion (p < 0.01).

**Conclusion:**

The ischiofemoral ligament primarily restricted the internal rotation of the hip joint. The piriformis and obturator internus may restrict internal rotation at 0° and 60° of flexion.

## Background

Total hip arthroplasty (THA) is one of the most successful procedures for reducing pain and improving function in patients with hip osteoarthritis. The number of patients undergoing THA is increasing and will likely continue in the future. However, dislocation and loosening after primary THA are serious adverse events. The most common causes of revision THA in the United States are hip dislocation and instability [1, 2]. Endogenous factors related to the patients, the surgeon’s skills, and the prosthesis design are factors that induce dislocation [3], as well as mechanical factors, such as the accuracy of the prosthesis setting and soft-tissue balance [4]. However, the optimal soft-tissue balance to prevent hip dislocation has not yet been determined.

The dislocation rate for patients who underwent a posterior surgical approach with capsular ligament repair was lower than patients without capsular ligament repair [5, 6]. These studies suggested that preserved soft tissues, including the capsular ligament and external rotators, help prevent excessive internal rotation and dislocation. The capsular ligaments of the hip are composed of the iliofemoral, ischiofemoral, and pubofemoral ligaments. Anatomically, the ischiofemoral ligament inserts into the ischium and posteroinferior areas of the acetabular rim and attaches to the posterior intertrochanteric line [7]. The ischiofemoral ligament controls the internal rotation and extension of the hip [8].

In previous cadaveric studies, the ischiofemoral ligament restricted primary internal rotation, particularly for flexion ≥ 30° and adduction [9]. However, these studies only examined the capsular ligament after removing the muscles around the hip joint. The functional role of the ischiofemoral ligament in restricting internal rotation has not been evaluated with a preserved capsular ligament and external rotator muscles. The synergistic effect of the ischiofemoral ligament and external rotator muscles for internal rotation restraint should be evaluated, as should the factors for preventing excessive internal hip rotation. This study determined the contribution of each external rotator muscle and the ischiofemoral ligament for preventing excessive internal hip joint rotation in relation to the hip flexion angle.

## Methods

### Cadaveric study

The study protocol was approved by the institutional review board of our institute. A total of ten normal hips (four right and six left hips) obtained from seven fresh-frozen cadavers (six males and one female) were enrolled. The age of the specimens at death ranged from 72 to 89 years (mean, 77.6 years). The pelvis was obtained by transecting the spine between the 4th and 5th lumbar spine, and the femur was cut at the mid-shaft level. All cadavers were thawed overnight at room temperature. The skin and subcutaneous adipose tissues were removed, but the muscles and capsular ligaments remained intact.

**Fig. 1:**
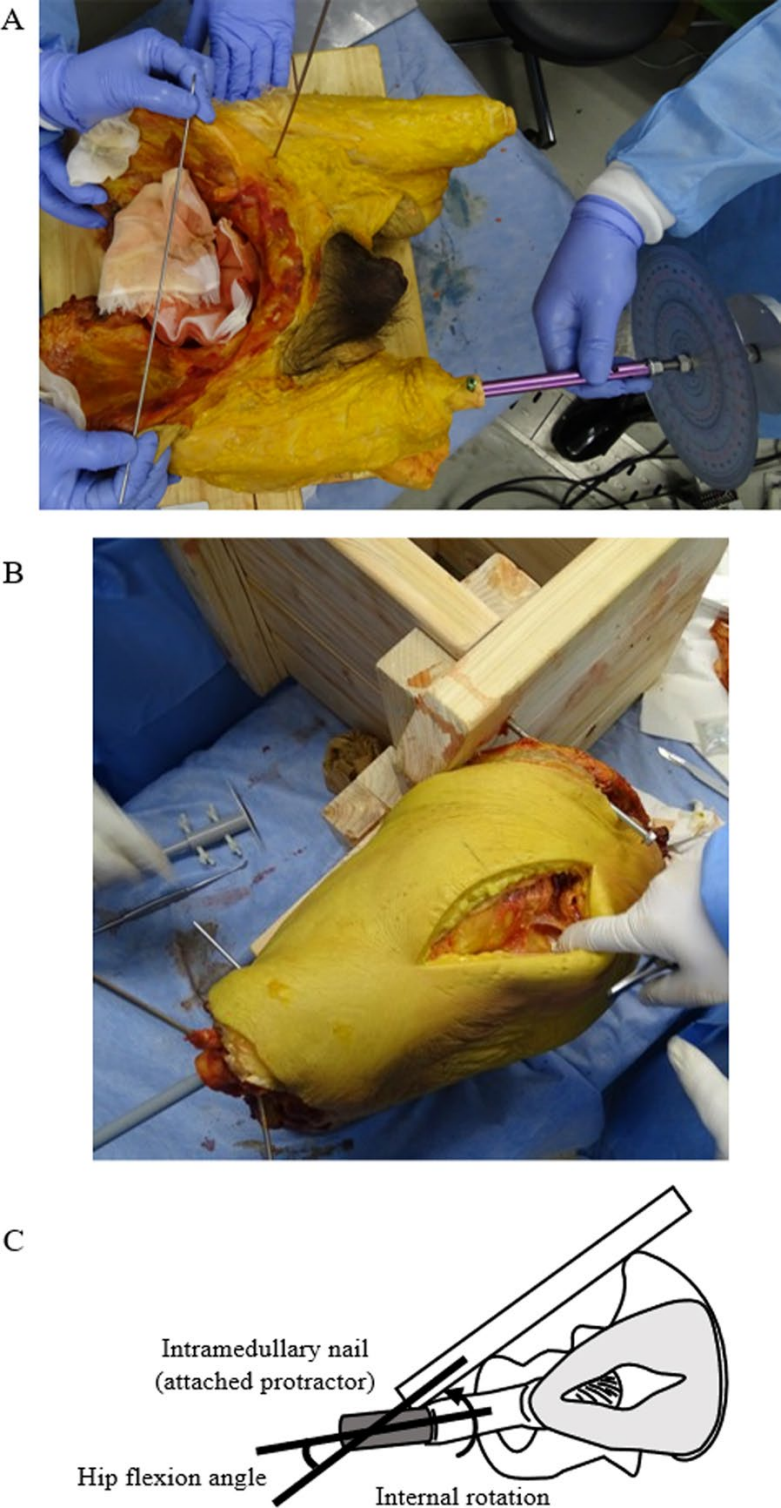
Photographs of the cadavers showing **A** the insertion of the Phoenix Ankle Arthrodesis Nail to the right femur (a protractor was attached to the nail) and **B** pelvic fixation (using a posterior approach). **c** A schematic model providing an overview of the study settings. The hip flexion angle was the angle between the femur and the anterior pelvic plane

**Fig. 2:**
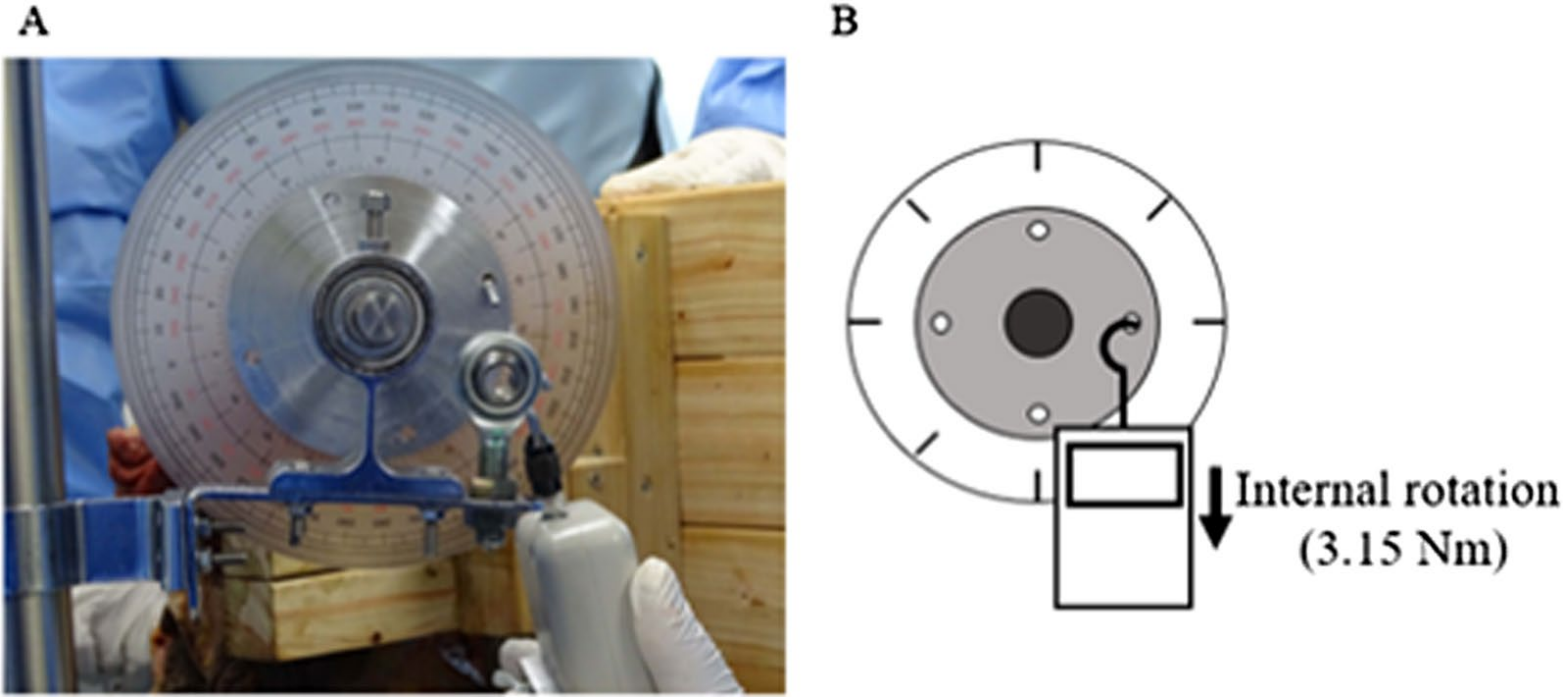
The angle-measurement method using a digital pull tension gauge. Photographs **A** and **B** demonstrate the methods used to measure the increasing angles using a digital pull tension gauge

The pelvis was fixed on a wooden plate by inserting K-wires at the anterior superior iliac spine and the ischial tuberosity. The pelvis was stabilized in the lateral decubitus position using a plate (Fig. 1). A Phoenix Ankle Arthrodesis Nail (Zimmer Biomet, IN, USA) was inserted into the distal femur and fixed with two screws (Fig. 1). A circular plate (90-mm diameter) with a protractor was fixed 350 mm away from the center of the femoral head (Fig. 1). The hip center was confirmed using fluoroscopy. With the capsular ligament and external rotators intact, the circular plate was pulled vertically in the direction of internal hip rotation at a constant force of 3.15 Nm applied by a digital pull tension gauge (DST-500 N, IMADA, Japan), then the protractor was photographed (Cyber-shot DSC-HX400V, Sony, Japan) (Fig. 2). The protractor indicated an angle between the preserved external rotators and the ischiofemoral ligament, defined as the baseline angle.

**Fig. 3:**
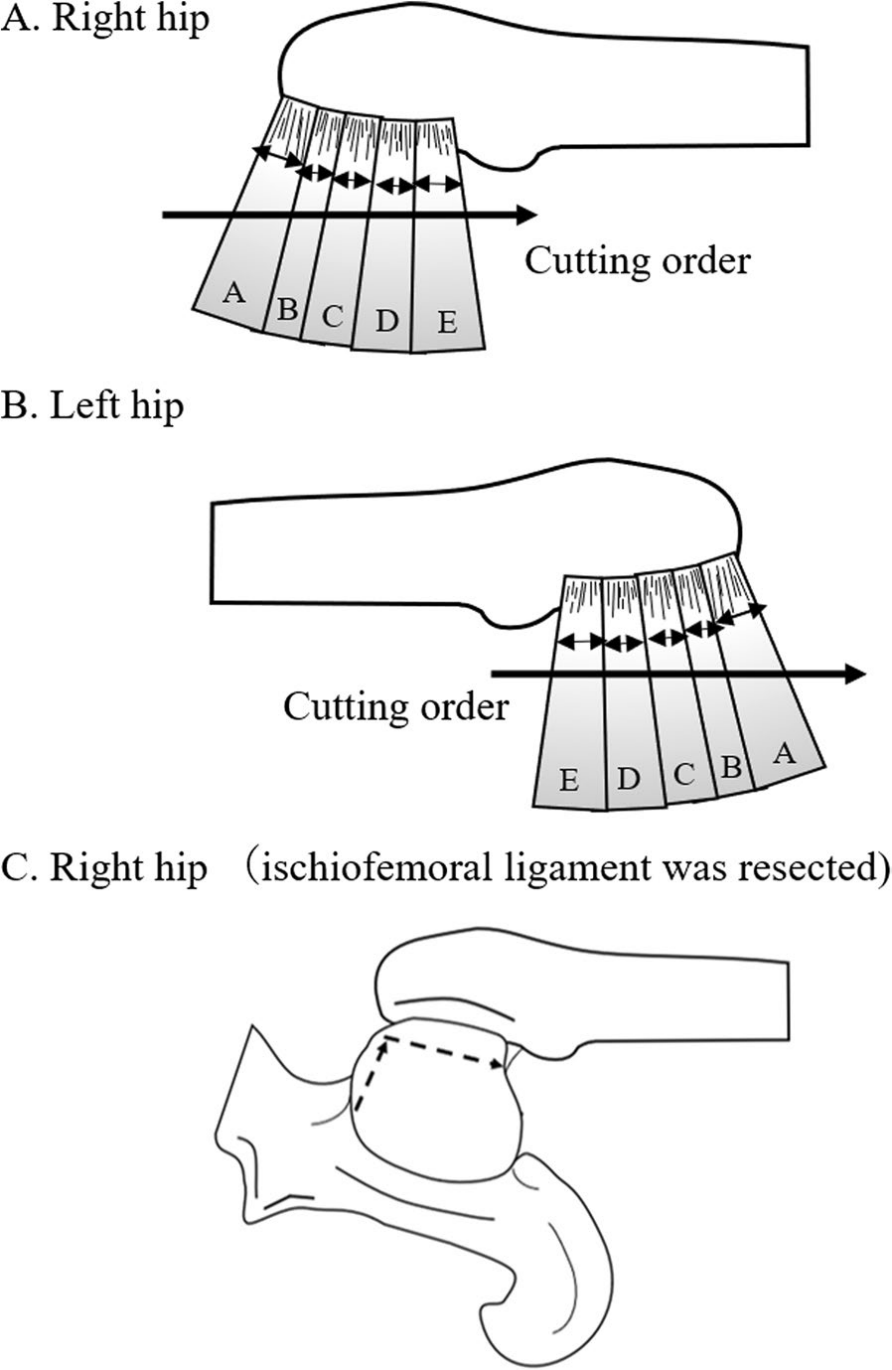
Schematic models of the stepwise dissections of the hip rotator muscles and ischiofemoral ligament in the posterior hip approach and reverse order. **A** The cutting order of the right hip via the posterior approach, indicated by the arrow. **B** The cutting order of the left hip in the opposite order from the right hip, indicated by the arrow. **c** The cutting order of the ischiofemoral ligament, indicated by the dotted arrow. A, piriformis; B, superior gemellus; C, obturator internus; D, inferior gemellus; E, obturator externus

The hip was dissected using a posterior approach [10]. The external rotators, including the piriformis, superior gemellus, obturator internus, inferior gemellus, and obturator externus, were individually identified. The external rotators were released from the piriformis to the obturator externus in the right hips and the reverse order (from the obturator externus to the piriformis) in the left hips (Fig. 3a, b). The protractor was photographed while a 3.15 Nm internal rotation torque was applied to the hip joint. Measurements were performed at 0°, 30°, and 60° of hip flexion, confirmed with a goniometer (Fig. 1). Finally, the ischiofemoral ligament was resected in an L-shape after resecting all the muscles (Fig. 3c). The acetabular labrum remained intact. The protractor measurement was repeated three times after each release step, and the average angle of the three measurements was recorded. An increase in the rotation angle was calculated as the difference between the baseline angle and the angle after each rotator muscle and the ischiofemoral ligament resection. All procedures were performed on the same day. The angle was measured from the photograph using ImageJ 1.51 software (National Institutes of Health, Bethesda, Maryland, USA). Two hip surgeons with more than ten years of experience independently measured the protractor angles.

### Statistical analyses

The results are presented as averages ± standard deviations. The coefficient of variation was calculated to determine the preciseness of the measurement using SPSS version 21.0 (IBM Corp., Armonk, NY, USA). Statistical differences in the angle based on the stepwise cuts of external rotators and the capsular ligament were performed using one-way analysis of variance with post hoc by Tukey–Kramer test. Statistical significance was set at p < 0.05.

## Results

**Fig. 4:**
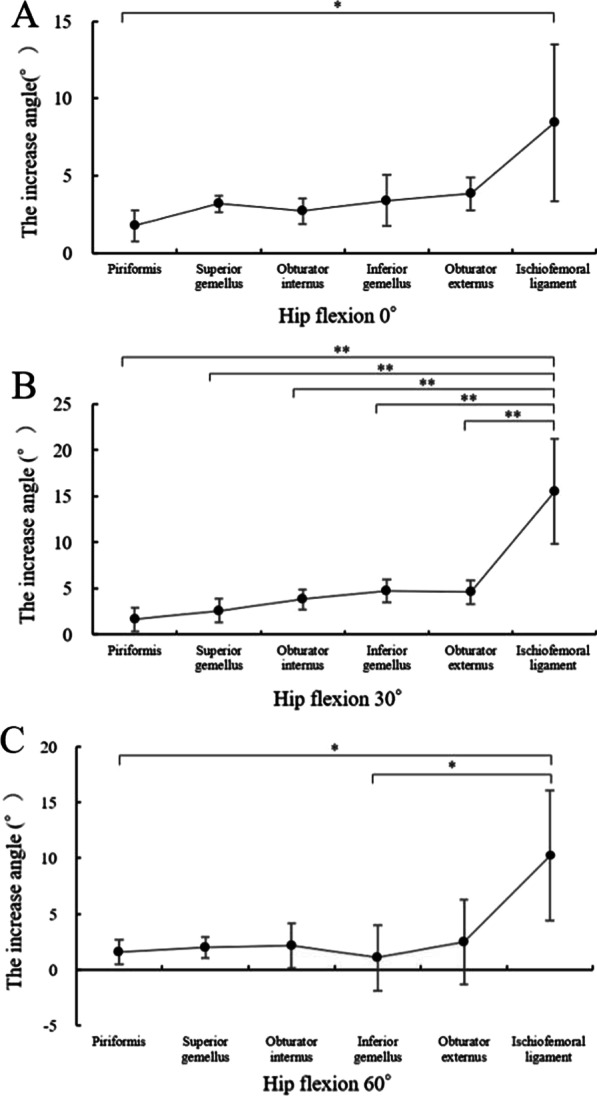
Increasing angles via the posterior approach from the piriformis to the obturator externus. The muscle name indicates the increased angle measured after muscle resection. *p < 0.05 and  **p < 0.01 by one-way analysis of variance with post hoc Tukey–Kramer test

**Fig. 5:**
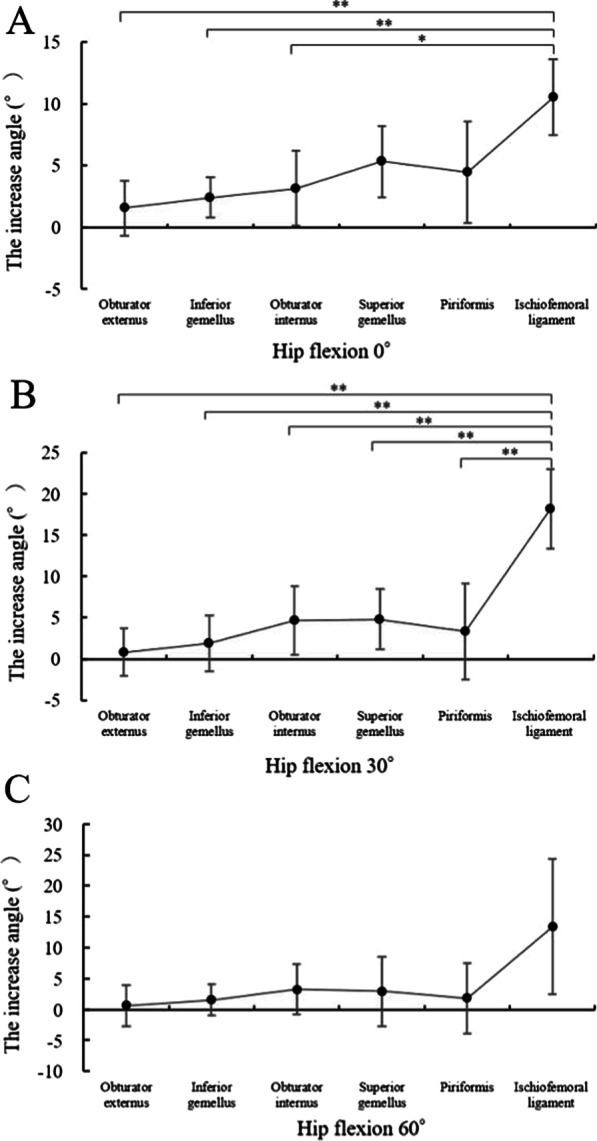
Increasing angles via the posterior approach from the obturator externus to the piriformis. The muscle name indicates the increased angle measured after muscle resection. * p < 0.05 and **p < 0.01 by one-way analysis of variance with post hoc Tukey–Kramer test

One hip was excluded because of femur fractures occurring during the procedures; nine hips were used for the analysis. In this study, no hips were dislocated during any of the procedures. The intra-class correlation coefficient of the measurements was 0.98 in the right hips and 0.90 in the left hips, and the angle measurement reproducibility was excellent; these values were almost in perfect agreement [11]. The increases in the angle after the stepwise resections of the external rotators did not differ regardless of the order (Figs. 4 and 5).

In the right hips, the results showed in Table 1 and Fig. 4. The angle measurements significantly differed between the piriformis and ischiofemoral ligament resections at 0° of flexion (p = 0.02; Fig. 4a), between each external rotator resection and the ischiofemoral ligament resection at 30° of flexion (all p < 0.01; Fig. 4b), and between the ischiofemoral ligament and piriformis resection and inferior gemellus resections at 60° of flexion (p = 0.04 and p = 0.02, respectively; Fig. 4c).

**Table 1:** Increasing angles via the posterior approach from the piriformis to the obturator externus. The muscle name indicates the increased angle measured after muscle resection. The data are presented as averages ± standard deviations

	Piriformis	Superior gemellus	Obturator internus	Inferior gemellus	Obturator externus	Ischiofemoral ligament
Hip flexion 0°	1.8° ± 1.0°	3.2° ± 0.5°	2.7° ± 0.8°	3.4° ± 1.7°	3.8° ± 1.1°	8.4° ± 5.1°
Hip flexion 30°	1.6° ± 1.3°	2.6° ± 1.3°	3.8° ± 1.1°	4.7° ± 1.3°	4.6° ± 1.3°	15.5° ± 5.7°
Hip flexion 60°	1.6° ± 1.1°	2.0° ± 0.9°	2.1° ± 2.0°	1.1° ± 2.9°	2.5° ± 3.8°	10.2° ± 5.8°

In the left hips, the results showed in Table 2 and Fig. 5. The angle measurements significantly differed between the ischiofemoral ligament and obturator externus, inferior gemellus, and obturator internus resections at 0° of flexion (p < 0.01, p < 0.01, and p = 0.01, respectively; Fig. 5a) and between each external rotator resection and the ischiofemoral ligament resection at 30° of flexion (all p < 0.01; Fig. 5b). None of the measurements differed at 60° of flexion.

**Table 2:** Increasing angles via the posterior approach from the obturator externus to the piriformis. The muscle name indicates the increased angle measured after muscle resection. The data are presented as averages ± standard deviations

	Obturator externus	Inferior gemellus	Obturator internus	Superior gemellus	Piriformis	Ischiofemoral ligament
Hip flexion 0°	1.6° ± 2.2°	2.4° ± 1.6°	3.2° ± 3.0°	5.3° ± 2.9°	4.5° ± 4.1°	10.5° ± 3.1°
Hip flexion 30°	0.8° ± 2.9°	1.9° ± 3.4°	4.7° ± 4.2°	4.8° ± 3.7°	3.3° ± 5.9°	18.1° ± 4.8°
Hip flexion 60°	0.6° ± 3.4°	1.5° ± 2.5°	3.2° ± 4.0°	2.9° ± 5.6°	1.8° ± 5.6°	13.3° ± 10.9°

## Discussion

This cadaveric study demonstrated that the ischiofemoral ligament primarily restricted excessive internal hip rotation, especially at 30° of hip flexion. The results also suggest that the external rotators interact synergistically, not individually, to restrict internal rotation at 0° and 60° of hip flexion.

The ischiofemoral ligament is one of the posterior capsular ligaments of the hip joint. The ligament is tense during internal rotation and adduction [12, 13]. When the hip is in deep flexion and internal rotation, ischiofemoral ligament tension increases and pulls the femoral head into the acetabulum. The tight ischiofemoral ligament protects against excessive internal hip rotation [9]. A previous study used a CT-based imaging technique to demonstrate that the strain on the hip joint capsular ligament was from an elongated ischiofemoral ligament during hip internal rotation. The ischiofemoral ligament length indicated that the maximum length occurred at a hip flexion of 30°, increasing from -15° to 30°, then decreasing from 30° to 90° [14]. These anatomical and biomechanical data were consistent with our findings that the ischiofemoral ligament was the primary contributor to hip stability by limiting the internal rotation between 0° and 60° of hip flexion, especially at 30°.

Recent reports have emphasized the importance of the ischiofemoral ligament and the external rotators in reducing dislocation risk after THA with a posterior surgical approach [15–17]. However, it was unclear which muscle resisted excessive internal hip rotation, especially at 0° and 60° of hip flexion, the ischiofemoral ligament was not stretched. In this study, when cutting from piriformis to obturator externus, the the obturator internus and the obturator externus were important for restricting internal rotation during hip flexion at 0° and 60° (Fig. 4a, c). In contrast, the reverse order resection indicated that the piriformis restricted internal hip rotation only at 0° of hip flexion (Fig. 5a). These results could be explained by changes in the moment arm and the force direction of the external rotators due to the hip flexion angle.

The structural property of the hip external rotators suggested that force generation was maximized to act as a single axis of rotation because its pennation angle was almost parallel [18]. Because of this structural property, the hip external rotators compressed the hip joint along a single axis. However, reports indicated that the moment arm of the external rotators changes depending on the hip flexion angle, which in turn changes the force direction. The moment arm of the piriformis muscle decreased as the hip flexion angle increased and switched from an external to an internal rotation moment arm at approximately 65° [19]. As a result, the piriformis did not restrict internal rotation at hip flexion angles greater than 60°. However, other reports indicated that the obturator internus and externus restricted internal rotation, even at 90° of hip flexion [19, 20]. Therefore, the piriformis functions as an external rotator at 0° of hip flexion and not at 60° due to decreased external rotation of the moment arm. Furthermore, the contributions of the obturator internus to restricting internal rotation at 60° of hip flexion might be more important than the piriformis.

Another important function of the external rotators is dynamic stabilization of the hip joint and performing pelvic and trunk rotational activities while walking [21]. External rotators might act synergistically in vivo to stabilize the hip joint at any hip flexion angle. An anatomical study of external rotators reported that the superior gemellus, obturator internus, inferior gemellus, and obturator externus were essentially fused [18]. Thus, these external rotators can interact with each other and function as one muscle. The authors considered the possibility that if one muscle was resected, the remaining muscles could compensate to some extent. These findings could explain why the restriction of internal rotation among external rotators in our study did not differ.

There were limitations to the present study. First, the sample size was small. Second, the study used cadavers. Therefore, the results could have been influenced by the fact that physiological muscle contraction, including the external rotators, was not possible. Further, the average age of the cadavers in this study was high, and the impact of age-related muscle weakness on the results was debatable. Accumulation of advanced glycation end products in the extracellular matrix with aging is associated with a decline in muscle force transmission in vivo [22], and the strength of the external rotators might not be equal between adolescents and older individuals in vivo. In contrast, a previous study comparing the stiffness of cadaveric pelvic floor muscles and obturator internus as controls demonstrated no difference in obturator internus muscle stiffness between adolescents and older individuals [23]. Therefore, age might have a limited effect on studies like ours investigating the prevention of internal hip rotation of external rotators using cadavers. Third, it was not possible to measure 90° of hip flexion due to the interference of the pelvic fixation device.

## Conclusions

We suggest that excessive internal hip joint rotation is primarily prevented by the ischiofemoral ligament, especially at 30° of hip flexion. The piriformis and obturator internus together might also restrict internal rotation at 0° and 60° of hip flexion.

## Data Availability

All data generated or analyzed during this study are included in this published article.

## Abbreviation

THA: Total hip arthroplasty
